# Improving xylose utilization by recombinant *Zymomonas mobilis* strain 8b through adaptation using 2-deoxyglucose

**DOI:** 10.1186/1754-6834-7-19

**Published:** 2014-02-01

**Authors:** Ali Mohagheghi, Jeff Linger, Holly Smith, Shihui Yang, Nancy Dowe, Philip T Pienkos

**Affiliations:** 1 National Bioenergy Center, National Renewable Energy Laboratory, 15013, Denver West Parkway, Golden, CO 80401, USA

**Keywords:** Zymomonas, Xylose, 2-deoxyglucose, Pretreated corn stover, Adaptation, Simultaneous saccharification and fermentation, Next generation sequencing (NGS)

## Abstract

**Background:**

Numerous attempts have been made to improve xylose utilization in *Z. mobilis* including adaptive approaches. However, no one has yet found a way to overcome the reduced xylose utilization observed in fermentations carried out in the presence of glucose as well as the inhibitory compounds found within pretreated and saccharified biomass. Our goal was to generate *Z. mobilis* strains that are more robust than the wildtype strain with increased productivity in fermenting the glucose and xylose present in PCS. Through adaptation in the presence of 2-deoxyglucose, we have generated *Zymomonas mobilis* strain #7, which is better suited to utilizing xylose in pretreated corn stover (PCS) fermentations in the presence of both glucose and model inhibitory compounds of acetate and furfural. Strain #7 over performed the parent strain 8b both on simultaneous saccharification and fermentation (SFF) of PCS and fermentation of saccharified PCS slurry. At 65% neutralized PCS liquor level, strain #7 used 86% of the xylose present in the liquor while strain 8b was not able to ferment the liquor under similar conditions. Similarly, under SSF process conditions with 20% total solids loading of PCS, strain #7 used more than 50% of the xylose present, while strain 8b did not utilize any xylose under this condition. We have further identified genetic alterations in strain #7 in relation to the parental strain 8b that may be responsible for these phenotypic enhancements.

**Results:**

We performed an extended lab-directed evolution of *Z. mobilis* strain 8b in the presence of acetate and a non-hydrolyzable glucose analogue 2-deoxyglucose. Following the adaptation, we identified and characterized numerous candidate strains and found a dramatic increase in xylose usage not only in shake flask, but also in a controlled PCS fermentation. We re-sequenced the genomes of evolved strains to identify genetic alterations responsible for these improved phenotypes, and identified two mutations that may be key to the improved xylose usage in these strains.

**Conclusion:**

We have generated *Z. mobilis* strain #7, which can ferment xylose efficiently in the presence of toxins present in pretreated corn stover. Genetic alterations responsible for the improvement have been identified.

## Background

The revised Renewable Fuel Standard (RFS2) set forth by the Energy Independence and Security Act (EISA) of 2007 mandates producing increased volumes of renewable fuels to be blended with liquid transportation fuels. Cellulosic ethanol, considered to be an advanced biofuel, is expected to play a significant role in achieving the RSF2. Accordingly, continued pursuit of commercially viable cellulosic ethanol production remains at the forefront of biofuels research despite rising interest in hydrocarbon biofuels.

A particularly attractive ethanologen candidate for producing cellulosic ethanol is the Gram-negative alphaproteobacterium, *Zymomonas mobilis* (*Z. mobilis*). Using the Entner-Doudoroff (ED) pathway [1,2], *Z. mobilis* is capable of producing ethanol at a faster rate than many yeasts [3-5], utilizes very high sugar concentrations [4], and has a naturally high resistance to high ethanol concentrations (up to 13% (v/v)) [6]. In addition, the availability of the *Z. mobilis* genome sequence [7,8] facilitates strain improvement efforts. The major drawback of *Z. mobilis* had been its limited natural range of sugar substrates, consisting only of glucose, fructose, and sucrose [1,5,9]. To overcome this limitation, *Z. mobilis* has been engineered to use xylose [2] and arabinose [10], the second and third most abundant sugars found within pretreated and saccharified corn stover hydrolysate [11].

Numerous attempts have been made to improve xylose utilization in *Z. mobilis*, including adaptive approaches [2,12,13]. However, one problem that has not yet been sufficiently addressed is the reduced xylose usage under real-world conditions (that is, in the presence of glucose as well as the inhibitory compounds found within pretreated and saccharified biomass). For example, our in-house xylose-fermenting strains, including *Z. mobilis* 8b [2], differ dramatically in their ability to utilize xylose in a pure sugar medium compared to pretreated corn stover (PCS) slurry-containing medium, so attempts to improve xylose usage in pure sugars does not necessarily translate to better fermentative performance when using commercially relevant feedstocks. Two compounds that are commonly present in PCS are acetate and furfural, both of which are very toxic to *Z. mobilis*[14]. To generate *Z. mobilis* strains that could utilize xylose efficiently in the presence of glucose and acetate, we performed an extended laboratory-directed evolution of *Z. mobilis* strain 8b in the presence of acetate and a non-hydrolyzable glucose analog, 2-deoxyglucose, following a similar strategy employed with *Saccharomyces cerevisiae*[15]. After a prolonged adaptation process, we identified and characterized numerous candidate strains and found a dramatic increase in xylose usage not only in the shake flask, but also in a controlled PCS fermentation. We have recently employed a re-sequencing approach to the genetic loci contributing to acetate tolerance for a *Z. mobilis* mutant generated through random chemical mutagenesis and turbidostat enrichment [16]. We used the same approach to identify genetic alterations responsible for these improved phenotypes, and identified two mutations that may be key factors for improved xylose usage in these strains.

## Results and discussion

**Figure 1:**
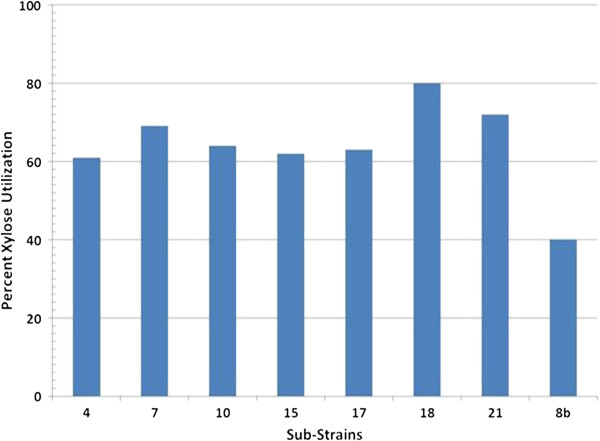
**Xylose utilization of different strains in rich medium supplemented with xylose (RMX) (10%), pH 5.8 and temperature 33°C, in 125-mL shake flasks.** Three strains with higher xylose utilization were selected for Bioscreen C testing: #7, #18, and #21.

We sought to improve xylose utilization of our recombinant *Z. mobilis* strain 8b in the presence of acetate through serial transfer in the presence of the non-hydrolyzable glucose analog, 2-deoxyglucose, based on the hypothesis that xylose is not able to compete effectively with glucose for transport into the cell. Briefly, 8b was grown continuously and serially transferred when optical density (OD) was above 1 with 10% (v/v) in rich medium (RM) supplemented with xylose, RMX (5%), supplemented with 5.0 g/L acetate and 0.5 to 2.0 g/L 2-deoxyglucose. Acetate was included in our enrichment strategy because it is produced during pretreatment of biomass and it has been shown that xylose-grown cells are more sensitive to this compound than are glucose-grown cells [17]. Thus we chose a strategy that would enrich for spontaneous mutants with alterations in one or both independent phenotypes. After 3 months of daily serial transfer, 20 colonies were isolated and revived as monocultures in RMX. Subsequently, the 20 isolates were screened for improved xylose utilization by growing in RMX (10%) in a shake flask with an initial pH of 5.8 and a temperature of 33°C for 48 hours. There was a variation of xylose usage among the strains ranging from approximately 60% to 80% compared to 8b, which used 40% of the xylose (Figure 1). In subsequent screening trials, three isolates, sub-strains #7, #18, and #21, had a higher xylose utilization rate than 8b when grown in RMX (10%) (data not shown). In some cases, this improved xylose usage was near two-fold over the parental strain (8b) suggesting that our strategy was very effective at improving xylose utilization.

**Figure 2:**
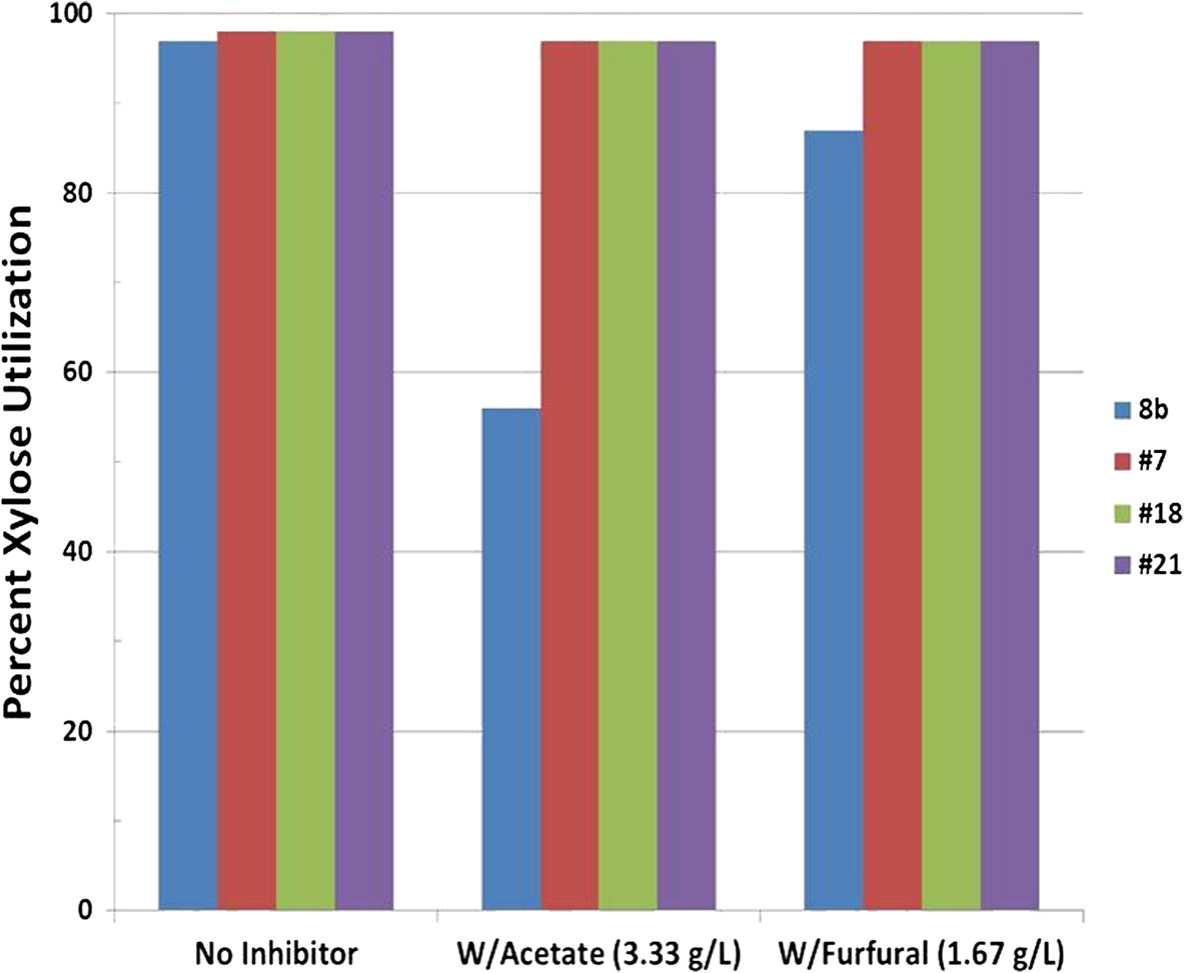
**Comparison of percent xylose utilization by strains 8b, #7, #18, and #21 in BioScreen C, initial pH 5.8, temperature 33°C, after 48 hours fermentation.** With Acetate (3.33 g/L); W/Furfural (1.67 g/L).

**Figure 3:**
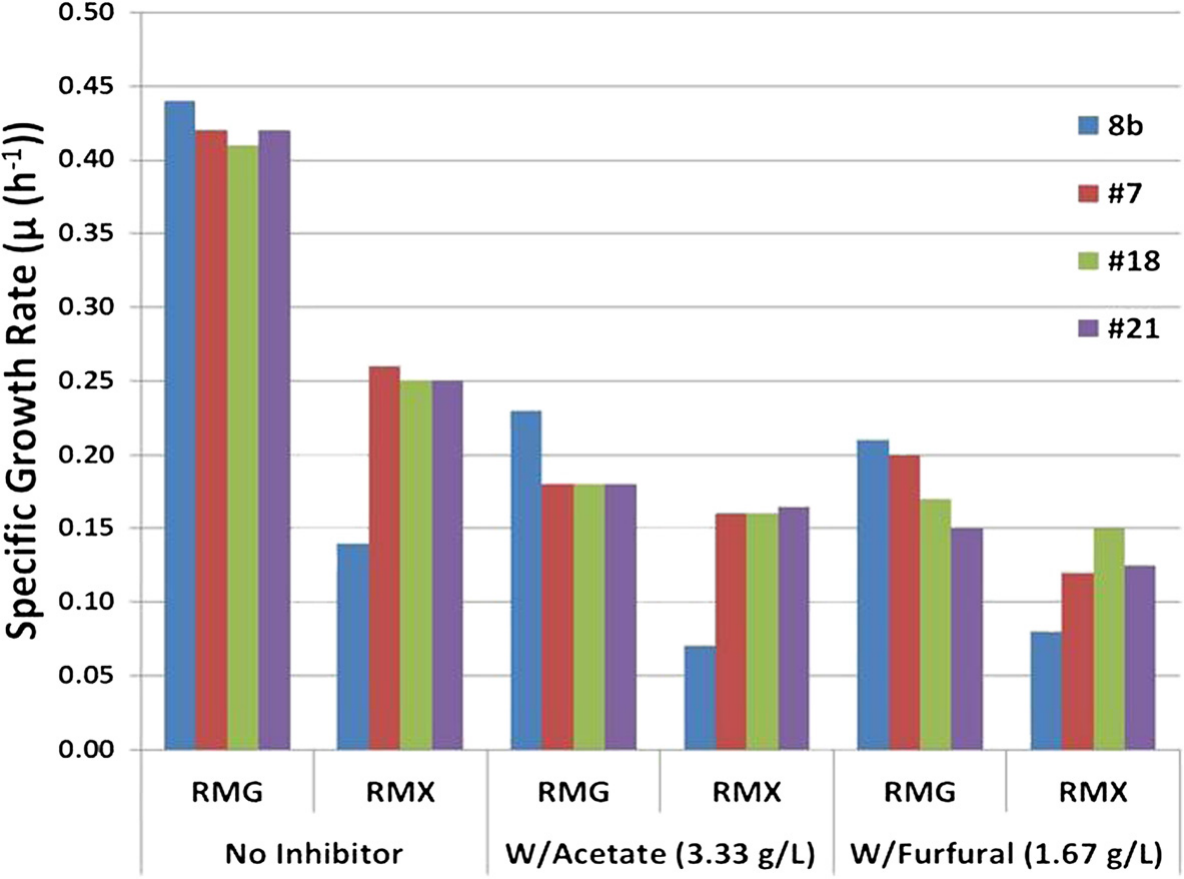
**Growth rate comparison of strains 8b, #7, #18, and #21 in rich medium supplemented with glucose (RMG) (2%) or with xylose (RMX) (2%) using BioScreen C, initial pH 5.8, temperature 33°C, after 48 hours fermentation.** With Acetate (3.3 g/L); W/Furfural (1.67 g/L).

We were also interested in understanding the ability of these new strains to utilize xylose in the presence of acetate, which was part of our selection strategy as well as the well-established inhibitory compound, furfural. Acetate and furfural represent two prominent inhibitors that are produced during pretreatment of biomass. Accordingly, evaluating the effect of these inhibitors was critical. We compared their performance using Bioscreen C with RMX (2%) supplemented with inhibitors. In the presence of inhibitors, it is clear that the adapted strains were much more effective at utilizing xylose, particularly in the presence of acetate (Figure 2). Interestingly, in the absence of inhibitors, as Figure 2 shows, there is very little difference in xylose usage among the strains at the end of fermentation but the rate may be faster for adapted strains, suggesting 8b only has difficulties in the rate of xylose utilization. Figure 3 shows the calculated maximum specific growth rate (μ_m_) of all strains in rich medium supplemented with glucose, RMG (2%), and RMX (2%) in the presence or absence of the inhibitory compounds, acetate and furfural. Interestingly, with glucose as the sole carbon source, 8b has an equivalent or greater growth rate than all three adapted strains, either in the absence or presence of inhibitory compounds. Conversely, the growth rate of the adapted strains surpasses the parental 8b strain in all media containing xylose. Based on these results and additional analyses (data not shown) of the adapted mutants, we concluded that #7 was the best performer of all candidate strains and the remainder of our research focuses on this strain.

**Figure 4:**
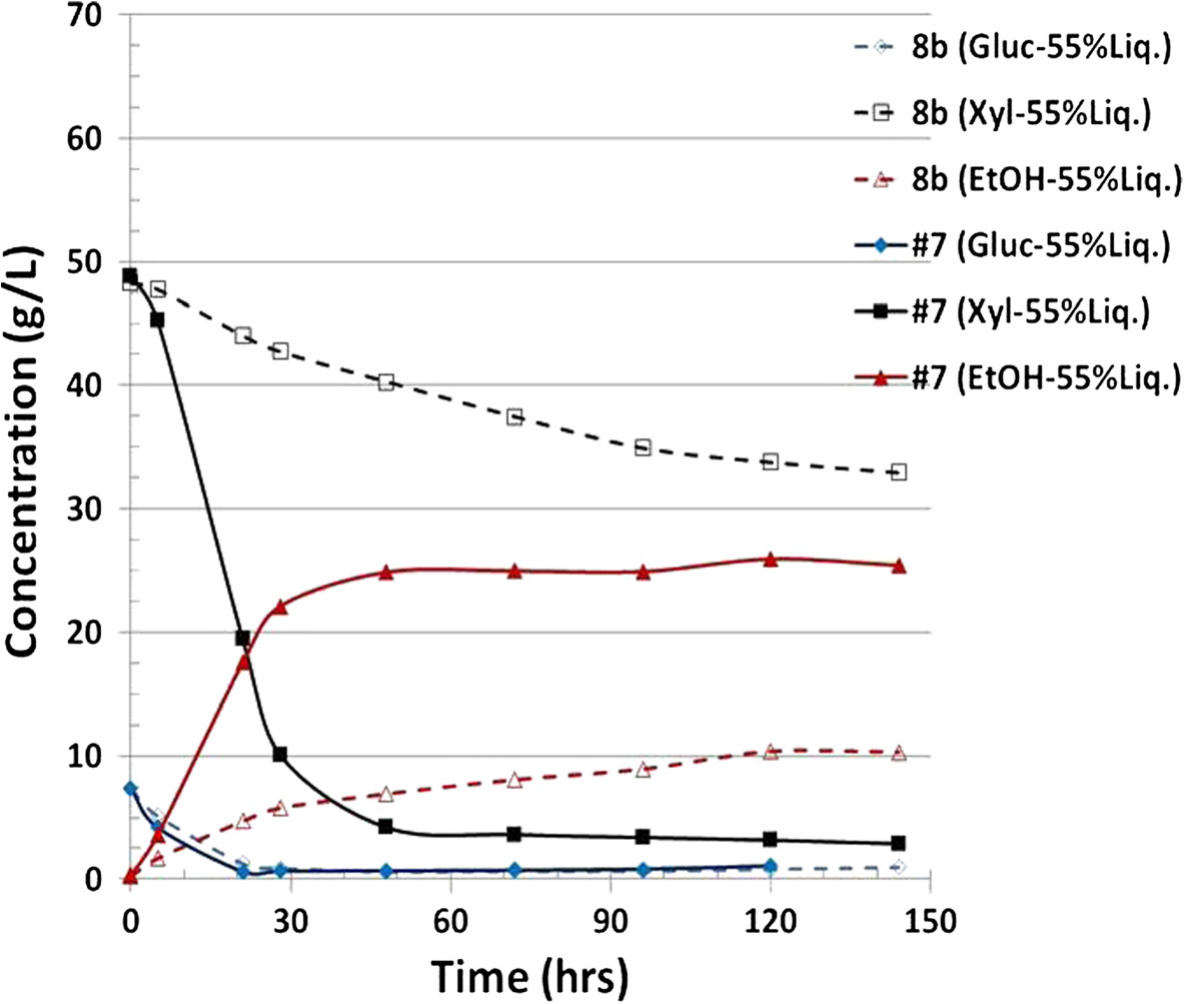
**Fermentation profile for strain 8b and #7 grown on 55% ammonia neutralized pretreated corn stover (PCS) liquor, pH 5.8, temperature 33°C.** Gluc, glucose; Xyl, xylose; EtOH, ethanol.

**Figure 5:**
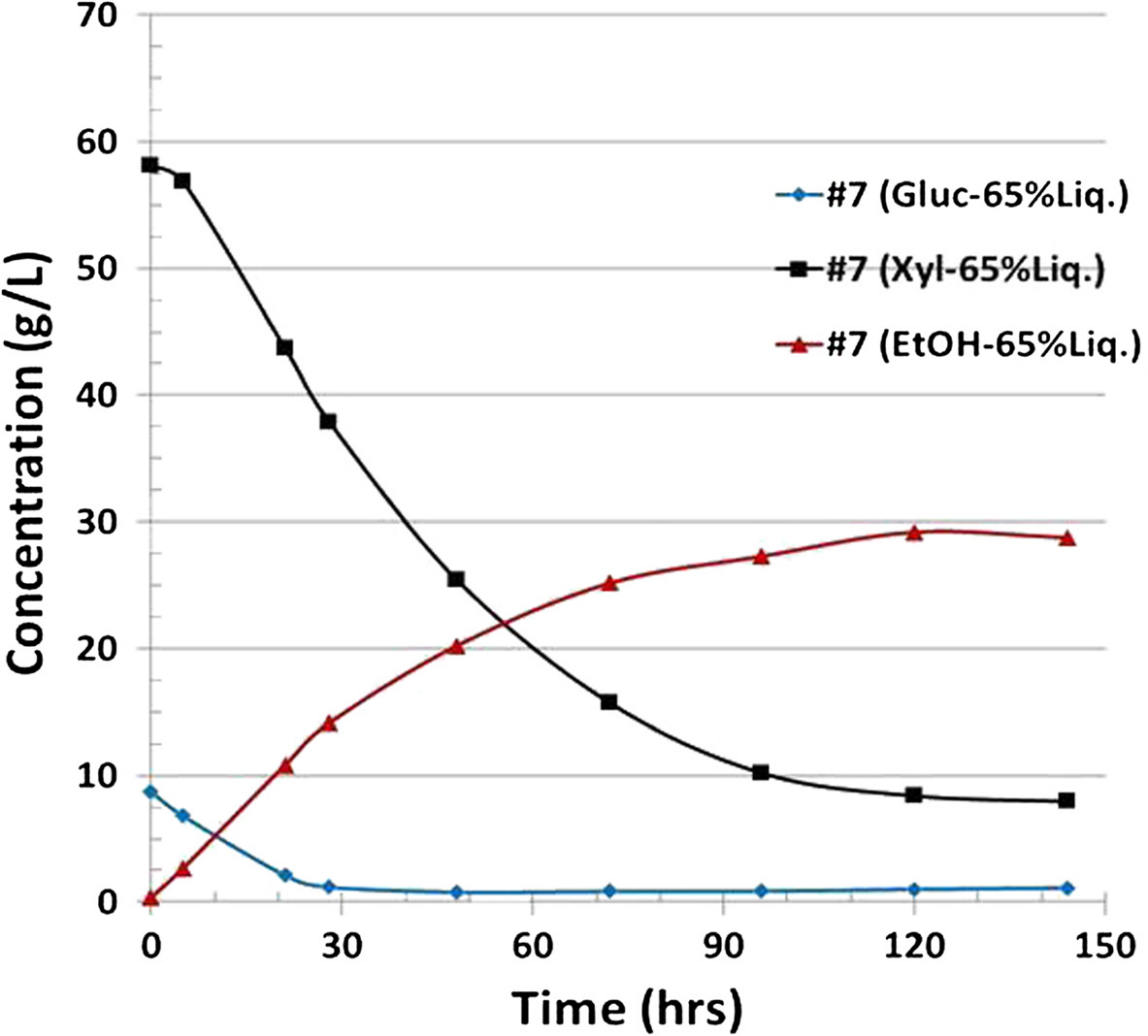
**Fermentation profile for strain #7 grown on 65% ammonia neutralized pretreated corn stover (PCS) liquor, pH 5.8, temperature 33°C.** Strain 8b was unable to grow in this medium, and as such, the data are not plotted. Gluc, glucose; Xyl, xylose; EtOH, ethanol.

Given that xylose usage in pure sugar medium is not necessarily an indicator of commercially relevant fermentative ability, we further assessed the fermentative performance of strain #7 in process-relevant conditions. Figures 4 and 5 summarize the fermentation of the saccharified slurry prepared by enzymatic hydrolysis of ammonia neutralized corn stover hydrolysate in both 55% and 65% (v/v) conditions. These concentrations correspond to total solids loading of 18% and 21%. Clearly, strain #7 outperformed 8b in the case of 55% liquor by drastically improving xylose usage and ethanol production. At the 65% loading, the difference between 8b and #7 was even more dramatic. Although the performance of #7 was partially inhibited by this higher concentration relative to the 55% (v/v) condition, it was still able to grow and utilize both glucose and xylose. Strikingly however, Strain 8b was entirely unable to grow in the 65% neutralized liquor (Figure 5). It is important to note that in fermentation, solids loading is a critical element in the overall cost of production because this parameter is a determinant of fermentation capacity and thus contributes to capital costs.

**Figure 6:**
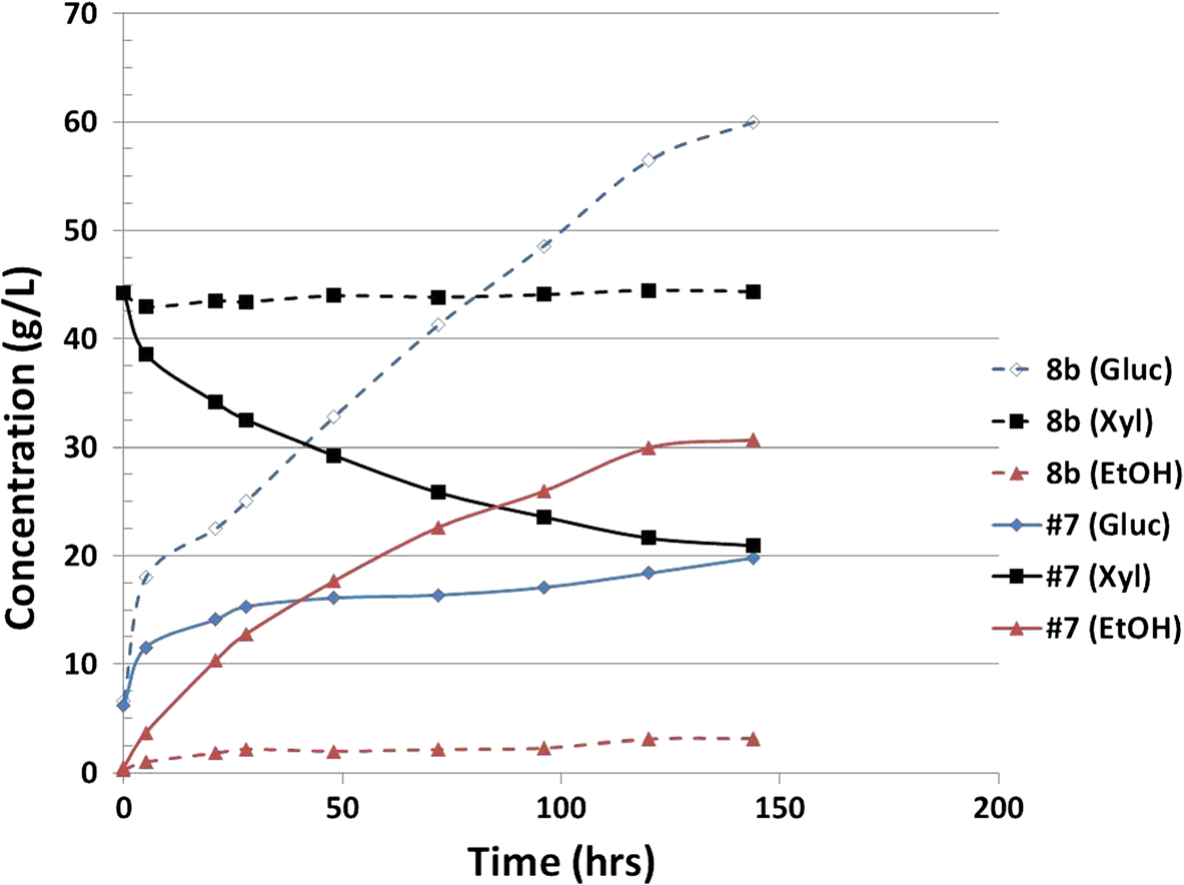
**Simultaneous saccharification and fermentation (SSF) profile for strain 8b and #7 on 17.5% total solids (TS) (W/V) whole slurry pretreated corn stover, pH 5.8, temperature 33°C.** Gluc: glucose; Xyl: xylose; EtOH: ethanol.

**Figure 7:**
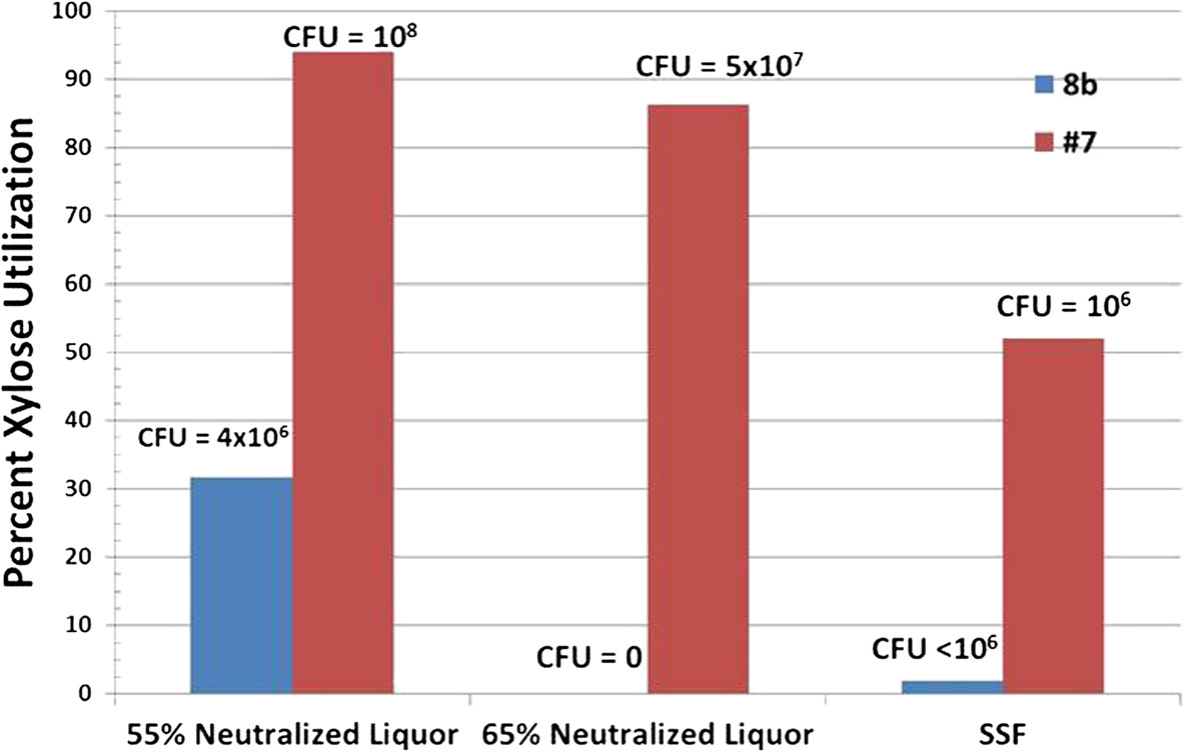
**Xylose utilization and cell number for strain 8b and #7 grown on 55% and 65% ammonia neutralized liquor and 17.5% total solids (TS) simultaneous saccharification and fermentation (SSF).** CFU, colony-forming units.

We further assessed the fermentative performance of strain #7 in a simultaneous saccharification and fermentation (SSF) configuration (Figure 6). Again, it was clear that strain #7 outperforms the parental strain 8b in glucose usage, xylose usage, and ethanol production under these process conditions. During the SSF process, 8b loses viability, which causes glucose accumulation. We have hypothesized that the failure of 8b to perform in SSF configuration is due to the impact of inhibitors, especially acetate, during the early stages of fermentation where xylose (released by hydrolysis of hemicellulose during the pretreatment process) is the major carbon source available to the cells. Figure 7 summarizes the xylose usage between the strains in the different conditions. In 55% (v/v) neutralized liquor, strain #7 utilized 92% of xylose compared with 32% for 8b. On 65% (v/v) neutralized liquor, strain #7 utilized around 86% of xylose whereas strain 8b was unable to grow. Similarly, in the SSF experiment (Figure 6) strain #7 had 52% xylose utilization whereas strain 8b had less than 5%.

To further understand the problems underlying the fermentation differences between 8b and strain #7, we sought to determine if 8b had reduced growth in response to these conditions. To assay for cell density in the presence of the dark-colored slurry, we performed cell counts at the end of fermentation by plating the culture using serial dilution protocol (Figure 7). The results are quite clear in that strain #7 had increased in cell density substantially more than 8b in all conditions. This is particularly true in the case of the 65% (v/v) liquor fermentation (Figure 6), where we were unable to detect any viable cells of strain 8b, and in the SSF trial (Figure 7), where we were able to detect very few. This suggests that the reduced fermentative capability of 8b in relation to strain #7 is a result of growth inhibition in the harsh conditions of these fermentations.

Given the vastly improved fermentative performance of strain #7 over 8b in regards to xylose utilization and the tolerance of inhibitors, we wanted to identify the underlying genetic basis for this improved phenotype. We used Next Generation Sequencing technologies (NGS) to re-sequence the genome of both 8b and strains #7 and #18. The results of this re-sequencing revealed that despite months of selective pressure adaption, strains #7 and #18 were genetically identical (and therefore likely to be re-isolates of a variant to come to dominate the adapted culture) and had acquired only two single-nucleotide polymorphisms (SNPs) in relation to 8b. The first resides in the intergenic region between the divergently expressed *ZMO0959* and *ZMO0061*. According to National Center for Biotechnology Information (NCBI) *ZMO0959* encodes a monofunctional biosynthetic peptidoglycan transglycosylase, (NCBI) and *ZMO0961* encodes a cytochrome c class 1 enzyme. It is not yet apparent whether these SNP changes within the intergenic regions would affect gene transcription of either of these enzymes, and it is conceptually difficult to attribute the observed phenotypes to either or both of these genes. On the contrary, the second identified SNP occurs within the coding sequence of *ZMO0774,* which encodes a LysR transcriptional regulator. Although the exact functions of this gene product are unclear, it is likely that it exerts transcriptional regulation on genes important for surviving these conditions.

## Conclusion

Through adaptive approaches, we have successfully generated a *Z. mobilis* strain that is better suited to utilizing xylose in PCS fermentations in the presence of both glucose and model inhibitory compounds of acetate and furfural. We have further identified genetic alterations in strain #7 in relation to the parental strain 8b that may be responsible for these phenotypic enhancements. Future research will be designed to definitively correlate one or both of these genetic mutations to the enhanced xylose usage phenotype seen in the newly generated strains. It will be interesting to return to our frozen stocks taken throughout the enrichment period to determine when the two mutations occurred and to see if one or both is needed for the phenotypes observed. However our current hypothesis is that the mutation in the LysR-type transcriptional regulator (*ZMO0774*) might be the underlying mutation responsible. The mutation is found within the promoter region of *ZMO0774,* indicating that altered expression levels of this transcription factor may play a very central role in improved xylose utilization in the context of fermentations. LysR-type transcriptional regulators (LTTRs) represent the most abundant type of transcriptional regulator in the prokaryotic kingdom [18,19], thus making it nearly impossible to predict the function of this particular LTTR. It will be interesting to ascertain the global transcriptional differences in strains harboring this mutation to identify the end gene expression changes resulting in enhanced xylose usage in our strains. Further clues as to the general role of LTTRs in stress response have recently been identified by Xiao-hong Su’s laboratory. Specifically, they found ZMO0774 to be transcriptionally upregulated in the presence of furfural [20] and high concentrations of ethanol [18].

## Materials and methods

### Strain and growth media

*Z. mobilis* 8b was used in this study [2]. Where indicated, *Z. mobilis* strains were grown in RM 1% (w/v) yeast extract, and 0.2% KH_2_PO_4_, and carbon source in any given medium is indicated either by G (glucose) or X (xylose), followed by the percentage. For example, RMG (5%) indicates RM supplemented with 5% glucose.

### Pretreated PCS for whole slurry fermentation

Corn stover was pretreated in a 200-kg/d horizontal pretreatment reactor at a solids loading of 30% (w/w), temperature of 158°C, 0.0224 g H_2_SO_4_/g dry stover (15.9 mg/g pretreatment process + 6.5 mg/g for oligomer conversion), and an approximate residence time of 5 minutes. Oligomer conversion was performed in a separate reaction vessel for 20 minutes at 130°C. This lot of PCS was designated BPI091217, Drum 4. The pH was adjusted from 2.0 to 5.0 with 30% ammonium hydroxide and then transferred into a 2-L Schott bottle and diluted with water to a final concentration of 20% total solids, after accounting for enzyme addition [11].

### Enzymatic hydrolysis of pH-adjusted PCS

The pH-adjusted PCS slurry was enzymatically hydrolyzed with Cellic CTec 2 cellulase enzyme (Novozymes, Davis, CA, USA). The enzyme was added at 40 mg protein/g cellulose and incubated at 48°C and 200 rpm for 4 days. When hydrolysis was complete, the saccharified PCS slurry was transferred into the bioreactors for fermentation [21].

### Sub-strain #7 development

Sub-strain #7 was developed for faster xylose utilization by exposing the strain 8b to a non-metabolized analog, 2-deoxyglucose (0.5 to 2 g/L), in RMX (5%) media. The adaptation was done in 15 mL Falcon tubes with 10 mL media, initial pH of 5.8, and incubated with no shaking at 33°C. After adaptation on RMX supplemented with 2-deoxyglucose, the sub-strain was further adapted on RMX (5%) supplemented with 5 g/L acetate in the presence of 2 g/L 2-deoxyglucose. The final culture was streaked on RMX and 20 colonies were isolated and frozen at -70°C for further evaluation. After further evaluation, the number of sub-strains was narrowed down to three: #7, #18, and #21.

### Sub-strain screenings using Bioscreen C

We used a Bioscreen C analyzer (Growth Curves, Piscataway, NJ, USA) to measure growth rates of sub-strains in the presence of acetic acid and furfural [14]. The Bioscreen C instrument includes an incubator that provides constant temperature and mixing by linear shaking. Turbidity was measured using a wide-band (420 to 580 nm) filter, which is less sensitive to changes in the medium color. The analyzer can read two 100-well sterile honeycomb plates with covers for multiple measurements of microbial growth curves. Turbidometric measurements were made every 10 minutes during the course of the run using RM medium with 2% (w/v) glucose and 2% (w/v) xylose and containing the inhibitors furfural and acetate. Log-phase cultures of selected strains were obtained by inoculating overnight cultures in RMGX (8%:2%) at 33°C and allowing the cells to grow to an OD_600nm_ of approximately 4.0, which corresponds to 3% to 4% glucose remaining in the medium. Cells were then distributed to Bioscreen C microplates containing media as described to a starting cell density of OD_600nm_ = 0.05. Operation of the Bioscreen C and collection of turbidity measurements (OD_420–580nm_) were computer automated with EZ Experiment (Norden Logic Oy, Helsinki, Finland). Data were collected and exported to Microsoft Excel spreadsheets and then the growth rate was calculated.

### Seed cultures

*Z. mobilis* 8b and sub-strains #7, #18, and #21 were taken from cell stocks stored at -70°C. The pre-seed medium used was RMGX (8%:2%). The strains were revived by transferring 1 mL of the frozen stock into 9 mL of pre-seed medium in a 15-mL tube. The cultures were incubated at 33°C with no agitation and then sampled at 8 hours for an optical density reading at 600 nm using Spectronic 601 spectrophotometer (Milton Roy, Ivyland, PA, USA). The pre-seed culture was used to inoculate the seed flask with media composition of RMGX (8%:2%, w/v) or the batch seed fermentor, if needed, with media composition of RM (1×), 150 g/L glucose, 20 g/L xylose, and 1 g/L sorbitol. Temperature was controlled at 33°C and pH at 5.8 by 4 M KOH.

### Fermentation for evaluation of adapted strains

Fermentations to evaluate the strains on neutralized saccharified whole slurry and SSF were performed in BioStat-Q Plus fermentors at a 300-mL working volume using *Z. mobilis* strain 8b and evolved strain #7. RM was added to enzymatically hydrolyzed whole slurry (20% total solids) or to mixture of solids and enzyme in the case of SSF. The fermentors were inoculated at an OD_600nm_ of approximately 1.0 using a direct transfer procedure (10% v/v). The fermentation was conducted at 33°C, pH 5.8 controlled with 4 M KOH, and an agitation speed of 300 rpm. The fermentation was typically finished in 72 hours. Ethanol yield calculations were based on initial glucose, xylose, and fructose concentrations and differences between initial and final ethanol concentration.

### Cell viability

Cell viability was measured using serial dilution protocol in which the fermentation broth was diluted in RM up to 10^-7^, then plated on RMG (2%) plates. The plates were incubated at 30°C in an incubator oven and after colony formation the colony numbers were counted.

### Sampling/data collection/analysis

Samples from the fermentations were taken at various time points. The samples were centrifuged and filtered through a 0.2-μm syringe filter before placing in high pressure liquid chromatography (HPLC) vials. Analysis of fermentation samples has been described previously [22]. Briefly, the samples were analyzed for carbohydrates and organic acids by HPLC using the Shodex SP0810 carbohydrate column and the Biorad Aminex HPX-87H organic acids column. Sugar utilization, ethanol yield, and ethanol titers were calculated based on these HPLC data. We also determined the insoluble solids in the slurry and the density of the liquor for the initial and final time points [23].

### Genomic resequencing of the adapted strain through next-generation sequencing (NGS)

Genomic DNA was extracted using Qiagen DNAEasy Kit (Qiagen, Germantown, MD, USA) and sent to the University of Utah for next-generation sequencing using Illumina Hiseq2000 (Illumina, Inc. San Diego, CA, USA) after passing the quality requirements. The quality of FASTQ genome resequencing data delivered from the University of Utah was checked using the FastQC program; data passing the quality control were imported into CLC Genomics Workbench 5.1 for data analysis to identity the potential small genetic changes such as SNP and Indels.

## Abbreviations

CFU: colony-forming units; ED: Entner-Doudoroff; HPLC: high pressure liquid chromatography; LTTR: LysR-type transcriptional regulator; NCBI: National Center for Biotechnology Information; NGS: next generation sequencing; OD: optical density; PCS: pretreated corn stover; RFS2: revised renewable fuel standard; RM: rich medium; RMG: rich medium supplemented with glucose; RMX: rich medium supplemented with xylose; SNP: single nucleotide polymorphism; SSF: simultaneous saccharification and fermentation.

## Competing interests

The authors declare that they have no competing interests.

## Authors’ contributions

AM designed the experiments and conducted the main part of the experiments. HS conducted the fermentation comparison, SY extracted genomic DNA and analyzed the next-generation sequencing data, JL helped on genomic sequencing and led writing of the manuscript (NGS), ND led experimental design and supervised the fermentation, PTP conceived the idea, provided technical oversight and served as critical reviewer of the manuscript. All authors read and approved the final manuscript.
